# Is there a role for prophylactic mesh in abdominal wall closure after emergency laparotomy? A systematic review and meta-analysis

**DOI:** 10.1007/s10029-019-02060-1

**Published:** 2019-10-22

**Authors:** F. A. Burns, E. G. Heywood, C. P. Challand, Matthew J. Lee

**Affiliations:** 1 Academic Department of General Surgery, Sheffield Teaching Hospitals NHS Foundation Trust, Sheffield, S5 7AU UK; 2 Department of Oncology and Metabolism, The Medical School, University of Sheffield, Sheffield, S10 2RX UK

**Keywords:** Incisional hernia, Emergency laparotomy

## Abstract

**Background:**

Incisional hernias are a common complication of emergency laparotomy and are associated with significant morbidity. Recent studies have found a reduction in incisional hernias when mesh is placed prophylactically during abdominal closure in elective laparotomies. This systematic review will assess the safety and efficacy of prophylactic mesh placement in emergency laparotomy.

**Methods:**

A systematic review was performed according to the PROSPERO registered protocol (CRD42018109283). Papers were dual screened for eligibility, and included when a comparison was made between closure with prophylactic mesh and closure with a standard technique, reported using a comparative design (i.e. case–control, cohort or randomised trial), where the primary outcome was incisional hernia. Bias was assessed using the Cochrane risk of bias in non-randomised studies tool. A meta-analysis of incisional hernia rate was performed to estimate risk ratio using a random effects model (Mantel–Haenszel approach).

**Results:**

332 studies were screened for eligibility, 29 full texts were reviewed and 2 non-randomised studies were included. Both studies were biased due to confounding factors, as closure technique was based on patient risk factors for incisional hernia. Both studies found significantly fewer incisional hernias in the mesh groups [3.2% vs 28.6% (*p* < 0.001) and 5.9% vs 33.3% (*p* = 0.0001)]. A meta-analysis of incisional hernia risk favoured prophylactic mesh closure [risk ratio 0.15 (95% CI 0.6–0.35, *p* < 0.001)]. Neither study found an association between mesh and infection or enterocutaneous fistula.

**Conclusion:**

This review found that there are limited data to assess the effect or safety profile of prophylactic mesh in the emergency laparotomy setting. The current data cannot reliably assess the use of mesh due to confounding factors, and a randomised controlled trial is required to address this important clinical question.

## Introduction

Incisional hernia (IH) is a protrusion of intra-abdominal contents through a surgically related defect in the anterior abdominal wall [1]. IH is a common complication of midline laparotomy [2] and can have a significant negative impact on quality of life [3]. Surgical repair can be challenging, with significant associated morbidity including reoperation, and recurrence rates can be as high as 10% [4]. It is therefore imperative that the focus is on prevention of this surgical complication. Most significantly, emergency laparotomy has been associated with high rates of IH of up to 22% [5], making it an area where resources might be concentrated.

Implantable mesh has traditionally been used in the elective repair of IH, however, recent studies have focused on its potential prophylactic use. There have been promising results in the elective setting, with a significant reduction in IHs seen in multiple randomised studies [6, 7]. However, these studies have focused solely on elective patients. As much elective surgery has moved towards minimally invasive approaches, the majority of laparotomies are now performed in the emergency setting, which is associated with high rates of IH [8, 9]. Furthermore, the European Hernia Society has recently suggested that non-midline incisions should be used to reduce the risk of incisional hernia [10]. However, this is clearly not an option in emergency laparotomy.

This makes the use of prophylactic mesh in the emergency setting an interesting proposition, as it may prevent the development of IH. However, there are concerns over potential mesh complications including infection, chronic pain and small bowel fistula. It is plausible that these complications could be more common in the emergency setting due to the increased incidence of peritonitis, intestinal obstruction and clinical condition of patients requiring emergency laparotomy.

The aim of this review is to assess the incidence of IH and post-operative complications in patients undergoing mesh closure of laparotomy in the emergency setting.

## Methods

This review was conducted as per a pre-defined protocol, published in PROSPERO (CRD42018109283—accessible via http://crd.york.ac.uk/prospero). The Preferred Reporting of Systematic Reviews and Meta-Analyses (PRISMA) [11] and Meta-analysis Of Observational Studies in Epidemiology (MOOSE) guidelines [12] were used to report the findings of the study.

The review was designed to answer the question ‘in patients undergoing emergency laparotomy, does use of prophylactic mesh in abdominal wall closure compared to standard closure technique(s) affect the rate of incisional hernia as reported in studies using comparative designs (e.g., case–control, cohort or randomised trial)’.

*Inclusion criteria* comparative studies (case–control, cohort, randomised trial) reporting on the effect and complications of mesh implantation in patients undergoing emergency midline laparotomy for a gastrointestinal indication with comparison to a control ‘standard’ technique were eligible for inclusion. No limitations were placed on the length of follow-up. No time limit was placed on date of publication. The inclusion of comparative type studies was selected, as we wished to compare outcomes of two different interventions. As research in surgery often begins with smaller case–control or cohort type studies, we anticipated that there would be no randomised trial level evidence in this area. Therefore, study design criteria were left deliberately broad.

*Exclusion criteria* studies were excluded if they reported mesh repair of groin or parastomal hernias, non-emergency surgery, non-GI surgery, or the use of temporary mesh closure. Studies involving less than 30 participants, case reports or case series were excluded. Abstracts or conference proceedings were also excluded, as they were deemed to be of insufficient quality not having undergone a formal peer review process. Non-English language studies were also excluded due to resource limitations.

Embase, MEDLINE and Cochrane (CENTRAL) databases were searched, according to a pre-defined search strategy. Databases were searched from inception to the date of the search (1947–present), Medline (1879–present) and CENTRAL (1996–present). The search was run on 27/08/18 and a sample search strategy is presented in appendix A.

An online systematic review tool (http://www.covidence.co.uk) was used to screen search results. Authors FAB and EH independently screened the abstracts of the studies identified in the search. Following this, full texts of the included studies were independently assessed by FAB and EH to determine inclusion in the review. Conflicts between the reviewers were resolved through discussion with a third researcher (ML).

Data extraction from included studies was performed independently by authors FAB and EH. A data collection proforma was used and the following data were collected: number of participants, age, gender, BMI, ASA grade, smoking status, COPD, diabetes, immunosuppression, previous laparotomy, indication for surgery, aspects of operative procedure including upper vs lower GI, incidence of SSIs, IHs, enterocutaneous fistula, mesh explantation, protrusion of mesh, reoperation, mortality and IHs. Conflicts were resolved by discussion.

The primary outcomes were IH, wound dehiscence and surgical site infection (SSI). Secondary outcomes were post-operative complications and mortality. Diagnosis of IH was made either clinically or via post-operative imaging.

Risk of bias was assessed using the Risk Of Bias In Non-randomised Studies of interventions (ROBINS)-I assessment tool [13]. FAB and EH independently evaluated the risk of bias in each study, and conflicts were resolved through discussion with ML. Each manuscript included was checked to identify whether the study was registered, or if it had a pre-registered statistical analysis plan to allow assessment for selective reporting. The outcomes reported in each study were compared for overlap to identify whether similar types of data were presented for each study.

Statistical analysis was performed by FAB and ML using RevMan5 (Cochrane Collaboration). A meta-analysis was undertaken using a random effects model (using Mantel–Haenszel approach) to estimate risk ratio and 95% confidence intervals. Heterogeneity was calculated using the *I*^2^ statistic. Statistical significance was set at *p* ≤ 0.05 a priori. If more than ten studies were identified, publication bias would be assessed by visual inspection of funnel plots.

## Results

**Fig. 1:**
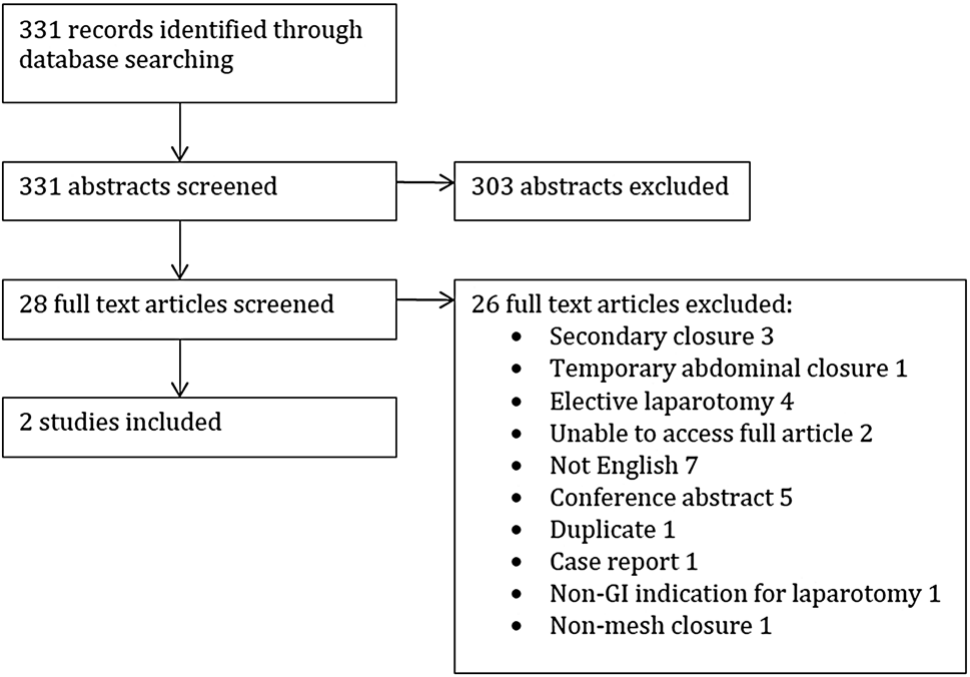
PRISMA flowchart

**Fig. 2:**
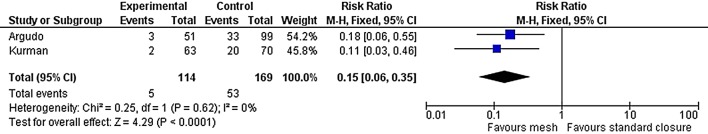
Forest plot showing risk ratio of IH in patients undergoing prophylactic placement of mesh for primary closure of laparotomy compared to those undergoing standard closure RR 0.15 (95% CI 0.06–0.35, *p* < 0.001)

A total of 331 studies were extracted from the search, of which 28 full text articles were screened. Of these, two were eligible for inclusion. Reasons for exclusion were mesh as secondary closure (*n* = 3) [14–16], mesh as temporary closure (*n* = 1) [17], elective laparotomy (*n* = 4) [18–21], unable to retrieve article (*n* = 2) [22, 23], not in English language (*n* = 7) [24–30], conference proceedings (*n* = 5) [31–35], duplicate (*n* = 1) [36], case report (*n* = 1) [37], non-GI indication for laparotomy (*n* = 1) [38] and non-mesh closure (*n* = 1) [39]. This is presented in the PRISMA flow diagram (Fig. 1). Two studies were identified as meeting the inclusion criteria [36, 40] (Fig. 2).

Bias assessment can be seen in Table 1. Both studies had a high risk of bias due to confounding factors as the operating surgeon decided on abdominal closure technique based on the presence of risk factors for IHs. Both studies had a low risk of bias in the selection of participants and classification of interventions and due to deviations from intended intervention. Argudo was judged to be at critical risk of bias; 115 patients were not included in the analysis due to missing data from loss to follow-up. There was a moderate risk of bias in both studies due to a lack of blinding. Bias due to selection of reported results was moderate in Argudo and low in Kurman. No funding source was reported for either study (Table 2).

**Table 1:** Bias assessment results for each study and domain using the ROBINS-1 tool

First author	Kurmann [36]	Argudo [40]
Bias due to confounding	Critical	Critical
Bias in selection of participants into the study	Low	Low
Bias in classification of interventions	Low	Low
Bias due to deviations from intended intervention	Low	Low
Bias due to missing data	Low	Critical
Bias in measurement of outcomes	Moderate	Moderate
Bias in selection of reported results	Low	Moderate
Overall bias	Critical	Critical

**Table 2:** Characteristics of studies

First author	Kurmann [36]	Argudo [40]
Year	2013	2014
Design	Case–control	Retrospective cohort
Patients	Undergoing laparotomy for peritonitis	Undergoing emergency midline laparotomy
Setting	University Hospital, Switzerland	University Hospital, Spain
Type of mesh	Non-absorbable, synthetic	Partially absorbable, synthetic
Mesh placement	Intraperitoneally	Mesh on fascia of medial rectus (onlay)
Securement of mesh	Single knot fascial sutures, endosurgical staples or combination	Fascial staples and polyprolene stitches
Comparator	Linea alba closure with running suture of PDS loop	Linea alba closure with running suture of PDS loop
Number of patients	133	150
Primary outcome	IH	IH
Definition of primary outcome	Abdominal wall gap with or without bulge in the area of post-operative scar, diagnosis clinically or on imaging.	Clinical diagnosis of IH, surgical intervention for IH or radiological diagnosis of IH

Both studies showed positive results of their studies, increasing probability of publication. No additional negative studies were identified. There is some concern that publication bias may affect findings here, as only positive studies were identified. Both studies reported incisional hernia as a primary outcome, with a range of secondary outcomes reported. The secondary outcomes seem clinically plausible and relevant. Both studies reported the same three secondary outcomes, and Argudo reported an additional 3 secondary outcomes. There are no published protocols or statistical analysis plans available to assess for selective reporting in a more robust manner.

Kurmann was a matched case–control study performed at a University Hospital in Switzerland, which included patients undergoing emergency laparotomy for peritonitis. This study compared the use of loop polydiaxanone (PDS) alone for abdominal closure versus mesh with PDS closure. The mesh used was a non-absorbable composite synthetic mesh (Parietine, Coviden; Parietex, Coviden or Dynamesh, Laubscher) implanted intraperitoneally fixed with single knot fascial sutures, endosurgical staples or a combination of the two. The primary outcome was IH defined as any abdominal wall gap with or without a bulge in the area of post-operative scar, diagnosed clinically or on imaging. Patients were followed up for a mean of 17 months (16 ± 4.7).

Argudo was a retrospective cohort study performed at a university hospital in Spain, in which patients underwent emergency midline laparotomy for peritonitis, obstruction, neoplasm, haemorrhage and ischemia. This study compared the use of PDS alone for abdominal closure versus a partially absorbable lightweight large pore synthetic mesh (Ultrapro, Ethicon) placed ‘onlay’ on the fascia of rectus abdominis, fixed with fascial staples and polypropylene stitches. The primary outcome was IH which was identified either by clinical diagnosis, surgical intervention for IH or by radiological diagnosis. Patients were followed up for 16 months (between 2 and 48).

Of the 299 patients included in both studies, 58 patients had IH. Kurmann et al. reported an IH rate of 3.2% (2/63) in the mesh group and 28.6% (20/70) in the control group (*p* < 0.001). Argudo et al. reported an IH rate of 5.9% (3/50) in the mesh group and 33.3% (33/100) in the control group (*p* = 0.0001). This is summarised in Table 3. Argudo found 15 of the incisional hernias (41.5%) required subsequent surgery, 1 in the mesh group and 14 in the control group. A meta-analysis of risk of IH favoured closure with prophylactic mesh, with a risk ratio of 0.15 (95% CI 0.06–0.35, *p* < 0.001). There was no heterogeneity noted.

**Table 3:** Outcomes

First author	Kurmann [36]	Argudo [40]
Follow-up	16 months	17 months
IH rate	Mesh: 3.2% Control: 28.6% (*p* < 0.001)	Mesh: 5.9% Control: 33.3% (*p* = 0.0001)
Surgical site infection	Mesh: 60.3% Control: 61.9% (*p* = 0.603)	Mesh: 26.3% Control: 17.6% (*p *= 0.13)
Enterocutaneous fistula	Mesh: 4.8% Control: 2.9% (*p *= 0.667)	Not reported
Mortality	Mesh: 9.5% Control: 7.1% (*p *= 0.008)	Mesh: 18.4% Control: 13.7% (*p* = 0.346)
Open abdomen	Mesh: 0 Control: 7.1% (*p* = 0.060)	Not reported
Reoperation	Mesh: 25.4% Control: 31.4% (*p* = 0.565)	Not reported

Surgical site infections were reported at a rate of 60.3% (30/63) vs 61.9% (39/70) (*p* = 0.603) for mesh and control groups, respectively, by Kurmann. In comparison, Argudo reported a rate of surgical site infection of 26.3% in the mesh group and 17.6% in the control group (*p *= 0.13). Kurmann reported that there was ‘no difference in the incidence of IH or SSI with respect to the different meshes and types of fixation’. Neither study reported the degree of peritoneal contamination at surgery.

Mortality was reported as 9.5% (6/53) at 30 days for the mesh group and 7.1% (5/70) in the control group (*p *= 0.756) in Kurmann’s study. Argudo et al. reported a mortality rate of 18.4% in the mesh group and 13.7% in the control group (*p *= 0.346). Kurmann et al. reported no patients in the mesh group having open abdomens, and 7.1% (5/70) of those in the control group (*p *= 0.060) and 25.4% (16/63) of those in the mesh group required reoperation, whilst 31.4% (22/70) in the control group required reoperation (*p *= 0.565). Enterocutaneous fistula was not reported by Argudo, and Kurmann et al. reported a rate of 4.8% (3/63) in the mesh group and 2.9% (2/70) in the control group (*p* = 0.667). Neither study reported the incidence of post-operative chronic pain.

## Discussion

This systematic review has presented the rate of IH following mesh closure of emergency laparotomies. It suggests that there may be a benefit of adding mesh to closure of emergency laparotomy to prevent incisional hernia formation.

Studies in elective settings show a significant reduction in IHs when there is prophylactic mesh placement [6, 7]. A recent trial of alternate closure methods found that there was no effect on IHs when emergency laparotomies were closed with interrupted fascial sutures compared with continuous fascial sutures [5].

Both included studies showed a significant reduction in IH rate when mesh was used in closure of emergency laparotomies compared to their standard practice of suture only. There was no statistically significant difference in the incidence of surgical site infection when mesh was compared to standard closure. Kurmann reported the incidence of enterocutaneous fistula, open abdomen and reoperation, and found no statistically significant difference between the mesh group and control group. Given infrequency of enterocutaneous fistula, it is probable that the study is not sufficiently powered to detect this at a meaningful rate. Open abdomen and reoperation were less common (although statistically insignificant) in the mesh group due to surgeon choice of suitable patients for mesh insertion. It is notable that the SSI rate in Kurmann was 60% vs 17% reported by Argudo. This may reflect differences in the degree of peritoneal contamination of patients entered into each study. When taken with the rest of the literature, this study suggests that addition of mesh in closure of emergency laparotomy might reduce the rate of IH formation, although the safety profile of this intervention has not been adequately described.

This is the first systematic review that has assessed the use of mesh closure following emergency laparotomy. IH remains a significant complication of abdominal incision and carries a considerable morbidity [4]. A recent randomised control trial demonstrated a significant reduction in incidence of IH when mesh was used to close elective laparotomies [41], which is supported by a 2017 meta-analysis [6]. However, evidence suggests an increase in chronic pain, post-operative seroma formation and delayed wound healing with mesh closure of elective laparotomies [6, 42]. Neither study included in this systematic review assessed the incidence of seroma or chronic pain. Given the public concern regarding mesh use, it is essential that future studies assess these potential complications. Neither of the studies included in this review detected any significant difference in complications between the mesh and control groups, including enterocutaneous fistula, surgical site infection or reoperation. Notably, the mesh group in one study had a significantly higher mortality rate than control groups which could be explained by selection bias in a non-randomised study.

This systematic review was limited by the small number of included patients (283) and only two studies met the inclusion criteria. No randomised trials were found that met the inclusion criteria. Selection bias into the intervention arm and lack of post-procedure blinding are further limitations. These may also lead to overestimation of the benefits of prophylactic mesh as only the highest risk patients received it (at the surgeon’s discretion). This same issue may lead to underestimation of the real-world rate of complications. There was significant heterogeneity between the studies, with multiple mesh types and placements between the two included studies, as well as differing pathology requiring surgery: Kurmann only included patients requiring laparotomy for peritonitis, which likely accounts for the much higher rate of surgical site infection. In addition, caution should be exercised in interpretation of the pooled effect. This is undertaken on unadjusted event rates in two studies of limited size, both of which have limitations in their design. This calculation should aid researchers in the field when calculating power and sample size for future studies.

Unfortunately, the data presented in this review do not provide strong enough evidence to either advocate routine mesh placement in this setting nor to advise against it. The studies included selected patients considered to be at high risk for IH after emergency laparotomy. This suggests there may be equipoise in this patient group. At least two further studies are required to explore this further, and should follow the IDEAL recommendations [43]. The first should be a large prospective cohort study which captures data on harms related to prophylactic mesh use, including seroma formation, chronic pain, and quality of life measures. This may also provide some indication of the optimum choice of mesh, as in contaminated or emergency cases, this remains uncertain [44].

Subject to acceptable risk profile of the interventions, a properly conducted randomised controlled trial with inclusion of only high-risk patients undergoing emergent laparotomy for peritonitis might be considered. The intervention should be a standardised selection of mesh with agreed standardised placement (e.g. Rives–Stoppa), with standardised suture fixation of the mesh. This should be compared to either the widely used mass closure, or small-bite closure which has been shown to be superior to mass closure in randomised trials [45]. The outcomes outlined above should be reported, as well as a robust health economic assessment.

## Conclusion

IH is a common complication of emergency laparotomy that is associated with significant morbidity. The current data does not reliably assess the use of mesh in preventing this complication, and a randomised controlled trial is required to address this important clinical question.

## Appendix

Search strategy:

Medline:

1. Exp surgical mesh/
2. Mesh.mp
3. Exp surgical wound dehiscence/
4. Exp emergencies/
5. Laparotom$.mp
6. Celiotom$.mp
7. Or/5–6
8. 4 and 7
9. 8 or 3
10. 1 or 2
11. 9 and 10

EMBASE:

1. Emergency laparotomy (including limited related terms)
2. Celiotomy (including limited related terms)
3. Mesh (including limited related terms)
4. Surgical mesh (including limited related terms)
5. 3 or 4
6. 1 or 2
7. 5 and 6

## References

[1] Ahn B-K (2012). Risk factors for incisional hernia and parastomal hernia after colorectal surgery. J Korean Soc Coloproctol.

[2] Fortelny RH (2018). Abdominal wall closure in elective midline laparotomy: the current recommendations. Front Surg.

[3] van Ramshorst GH, Eker HH, Hop WCJ (2012). Impact of incisional hernia on health-related quality of life and body image: a prospective cohort study. Am J Surg.

[4] Lowe JB, Lowe JB, Baty JD, Garza JR (2003). Risks associated with “components separation” for closure of complex abdominal wall defects. Plast Reconstr Surg.

[5] Peponis T, Bohnen JD, Muse S, et al (2018) Interrupted versus continuous fascial closure in patients undergoing emergent laparotomy: a randomized controlled trial. J Trauma Acute Care Surg 1 10.1097/TA.0000000000001970 29787547

[6] Borab ZM, Shakir S, Lanni MA, Tecce MG, MacDonald J (2017). Hope WW (2017) Does prophylactic mesh placement in elective, midline laparotomy reduce the incidence of incisional hernia? A systematic review and meta-analysis. Surgery.

[7] Jairam AP, Timmermans L, Eker HH (2017). Prevention of incisional hernia with prophylactic onlay and sublay mesh reinforcement versus primary suture only in midline laparotomies (PRIMA): 2-year follow-up of a multicentre, double-blind, randomised controlled trial. Lancet.

[8] Mingoli A, Puggioni A, Sgarzini G (1999). Incidence of incisional hernia following emergency abdominal surgery. Ital J Gastroenterol Hepatol.

[9] Saleem A-E-A, Abdallah H, Abdul Raheem O, Yousef M (2016). Rate of development of incisional hernia 1 year after urgent midline laparotomy. Al-Azhar Assiut Med J.

[10] Muysoms FE, Antoniou SA, Bury K (2015). European Hernia Society guidelines on the closure of abdominal wall incisions. Hernia.

[11] Moher D, Liberati A, Tetzlaff J (2009). Preferred reporting items for systematic reviews and meta-analyses: the PRISMA statement. PLoS Med.

[12] Stroup DF, Berlin JA, Morton SC (2000). Meta-analysis of observational studies in epidemiology: a proposal for reporting. Meta-analysis Of Observational Studies in Epidemiology (MOOSE) group. JAMA.

[13] Sterne JA, Hernán MA, Reeves BC (2016). ROBINS-I: a tool for assessing risk of bias in non-randomised studies of interventions. BMJ.

[14] Scholtes M, Kurmann A, Seiler CA (2012). Intraperitoneal mesh implantation for fascial dehiscence and open abdomen. World J Surg.

[15] Mozingo DW (2006). Multidisciplinary approach to abdominal wall reconstruction after decompressive laparotomy for abdominal compartment syndrome. Yearb Surg.

[16] Wang TY, Elliott R, Low DW (2013). Damage control abdomen: single-stage reconstruction using a vicryl mesh buttress. Ann Plast Surg.

[17] Fox N, Crutchfield M, LaChant M (2013). Early abdominal closure improves long-term outcomes after damage-control laparotomy. J Trauma Acute Care Surg.

[18] Caro-Tarragó A, Olona-Casas C, Olona-Cabases M, Guillén VV (2014). Retracted: impact on quality of life of using an onlay mesh to prevent incisional hernia in midline laparotomy: a randomized clinical trial. J Am Coll Surg.

[19] García-Ureña MÁ, López-Monclús J, Hernando LAB (2015). Randomized controlled trial of the use of a large-pore polypropylene mesh to prevent incisional hernia in colorectal surgery. Ann Surg.

[20] Iesalnieks I, Baladov M, Ikhlawi K (2015) Reinforcement of fascial closure by polyglactin-mesh to prevent abdominal wound dehiscence following open colorectal surgery. In: Diseases of the colon and rectum. Lippincott Williams & Wilkins, Philadelphia, pp E162–E162

[21] Timmermans L, Eker HH, Steyerberg EW (2015). Short-term results of a randomized controlled trial comparing primary suture with primary glued mesh augmentation to prevent incisional hernia. Ann Surg.

[22] Gainant A, Boudinet F, Cubertafond P (1989). Prevention of postoperative wound dehiscence in high risk patients. A randomized comparison of internally applied resorbable polyglactin 910 mesh and externally applied polyamide fiber mesh. Int Surg.

[23] Yüce K (1994). Retention mesh: an alternative to retention sutures. Eur J Surg.

[24] Argudo N, Pilar Iskra M, Pera M (2017). The use of an algorithm for prophylactic mesh use in high risk patients reduces the incidence of incisional hernia following laparotomy for colorectal cancer resection. Cirug Española (English Edition).

[25] de la Portilla F, de la Portilla F, Flikier B (2008). Estudio aleatorizado sobre la utilización de mallas reabsorbibles para la prevención de la evisceración en la cirugía colorrectal. Cirug Española.

[26] Haeder L, Jähne J (2013). Prophylactic mesh implantation in high-risk patients undergoing laparotomy. Chirurg.

[27] Paye F, Rongère C, Gendreau D, Lenriot JP (1992). Intraperitoneal resorbable mesh in the prevention of postoperative wound dehiscence.

[28] Robin-Lersundi A, Abella Álvarez A, Cruz Cidoncha A, López-Monclús J, Gordo Vi-dal F, García-Ureña MA (2013). Severe acute pancreatitis and abdominal compartment syndrome: treatment in the form of decompressive laparotomy and temporary abdominal closure with a expanded polytetrafluoroethylene mesh. Med Intensiva.

[29] Rodríguez-Hermosa JI, Codina-Cazador A, Ruiz B (2005). Factores de riesgo de dehiscencia aguda de la pared abdominal tras laparotomía en adultos. Cirug Española.

[30] Gürleyik G (2001). Factors affecting disruption of surgical abdominal incisions in early postoperative period. Turk J Trauma Emerg Surg.

[31] Jairam A, Lopez Cano M, Garcia Alamino J, Fitzgibbons FJ, Miserez M (2018). Hernia: world journal of hernia and abdominal wall surgery. 2018 International hernia congress.

[32] Jairam A, Timmermans L, Jeekel H (2017) 18th annual hernia Repair. In: Suppl 1. Springer, Berlin

[33] Dumanian G (2016) A novel mesh suture for laparotomy closure and hernia defect closure. In: 17th annual hernia repair, pp 1–138

[34] San Miguel C, Lopez J, Jimenez E (2017) Long-term outcomes after prophylactic use of onlay mesh in midline laparotomy. In: Hernia: oral and video presentations, pp 139–273 10.1007/s10029-018-1833-x 30288617

[35] San Miguel Mendez C, Garcia-Urena M, Blazquez-Hernando L (2018) First long-term results reported on prophylactic mesh closure of midline laparotomies. In: 2018 international hernia congress. Springer, pp 1–204

[36] Kurmann A, Barnetta C, Candinas D, Beldi G (2013). Implantation of prophylactic nonabsorbable intraperitoneal mesh in patients with peritonitis is safe and feasible. World J Surg.

[37] Mathes SJ, Harlan Stone H (1975). Acute traumatic losses of abdominal wall substance. J Trauma Injury Infect Crit Care.

[38] Brandt CP, McHenry CR, Jacobs DG (1995). Polypropylene mesh closure after emergency laparotomy: morbidity and outcome. Surgery.

[39] Mangus RS, Kubal CA, Tector AJ (2012). Closure of the abdominal wall with acellular dermal allograft in intestinal transplantation: intestinal transplant abdominal wall closure. Am J Transplant.

[40] Argudo N, Pereira JA, Sancho JJ (2014). Prophylactic synthetic mesh can be safely used to close emergency laparotomies, even in peritonitis. Surgery.

[41] Kohler A, Lavanchy JL, Lenoir U (2018). Effectiveness of prophylactic intraperitoneal mesh implantation for prevention of incisional hernia in patients undergoing open abdominal surgery. JAMA Surg.

[42] Payne R, Aldwinckle J, Ward S (2017). Meta-analysis of randomised trials comparing the use of prophylactic mesh to standard midline closure in the reduction of incisional herniae. Hernia.

[43] McCulloch P, Altman DG, Campbell WB (2009). No surgical innovation without evaluation: the IDEAL recommendations. Lancet.

[44] Majumder A, Winder JS, Wen Y (2016). Comparative analysis of biologic versus synthetic mesh outcomes in contaminated hernia repairs. Surgery.

[45] Deerenberg EB, Harlaar JJ, Steyerberg EW (2015). Small bites versus large bites for closure of abdominal midline incisions (STITCH): a double-blind, multicentre, randomised controlled trial. Lancet.

